# Infant exposure to Chinese famine increased the risk of hypertension in adulthood: results from the China Health and Retirement Longitudinal Study

**DOI:** 10.1186/s12889-016-3122-x

**Published:** 2016-05-25

**Authors:** Zhenghe Wang, Changwei Li, Zhongping Yang, Zhiyong Zou, Jun Ma

**Affiliations:** Institute of Child and Adolescent Health, School of Public Health, Peking University Health Science Center, No 38 Xue Yuan Road, Haidian District, Beijing, 100191 China; Department of Epidemiology, Tulane University School of Public Health and Tropical Medicine, New Orleans, LA USA

**Keywords:** Hypertension, Famine, Fetal Malnutrition, Developmental origin, Obesity

## Abstract

**Background:**

Early-life developmental adaptations in response to severe malnutrition may play a crucial role in susceptibility to hypertension. This study aimed to explore the associations between exposure to the Chinese famine (1959–1961) at fetal, infant and preschool stages during fetal life or childhood and the risk of hypertension in adulthood.

**Methods:**

We used the data of 1,966 adults born between 1956 and 1964 in selected families from the China Health and Retirement Longitudinal Study (CHARLS) national survey.

**Results:**

Prevalence of hypertension among adults in non-exposed, fetal-exposed, infant-exposed, and preschool-exposed cohorts was 18.9, 20.7, 28.7, and 23.4 %, respectively. In severely affected famine areas, only infant-exposed cohort had a significant increased risk of hypertension compared with non-exposed cohort (OR 2.12; 95 % CI 1.19, 3.79; *P* = 0.011), and the significance remained after adjusted gender, smoking, and drinking (OR 2.11; 95 % CI 1.18, 3.77; *P* = 0.012). After stratification by BMI and economic status, the risk of hypertension was higher for subjects with BMI ≥ 24 kg/m^2^(OR 2.09; 95 % CI 1.09, 4.01; *P* = 0.026) or high economic status(OR 2.26; 95 % CI 1.19, 4.31; *P* = 0.013) than those with BMI < 24 kg/m^2^(OR 1.65; 95 % CI 0.71, 3.83; *P* = 0.246) or low economic status (OR 2.18; 95 % CI 1.14, 4.18; *P* = 0.019) in infant-exposed cohort of severely affected famine areas. However, there was no consistent association observed in less severely affected area or other exposed cohorts in severely affected areas.

**Conclusions:**

Infanthood exposed to famine might increase the risk of hypertension in adulthood, and a postnatal ‘rich’ nutrient environment further increased the risk.

## Background

The Developmental Origins of Health and Disease (DOHaD) hypothesis postulates that adaptations in response to fetal malnutrition lead to metabolic and structural changes, which are beneficial for early survival, but may increase the risk of common diseases such as hypertension in adulthood [1–5]. The adverse risks of long-term consequences might further increase in a nutritionally rich environment in later life [2, 5]. Due to the ethical reason, there was no related study designed to validate the hypothesis in human beings. But historical famine provides a quasi-experiment setting for us to study the effect of severe malnutrition in early-life on inverse health outcomes, and thus provides direct evidence for the hypothesis in human beings. Several studies had explored the associations between famine exposure for six months at fetal stage in Dutch and blood pressure/hypertension in later life, which observed inconsistent results [6–8]. Another study from Leningrad Siege cohort in Russia found only adults who were exposed to famine in fetal stage had higher diastolic blood pressure than non-exposed group [9].

As one of the largest catastrophes in human history, the Chinese Great Famine began in the winter of 1959 and ended in the autumn of 1961, and has aroused much attention from scholars. The famine affected the entire mainland of China and caused 15 to 30 million of excess deaths, which was more devastating in most rural areas [10]. In recent years, a few studies used the data from epidemiologic surveys (e.g., 2002 China National Nutrition and Health Survey [11], Guangdong province health survey [12], and the China-U.S. Collaborative Project for Neural Tube Defect Prevention [13]) to explore the associations between the early life exposure to the Chinese great famine and blood pressure/hypertension in adulthood. But the associations revealed in these studies were inconsistent [11–13], and preschool exposure to famine was often ignored [13]. Furthermore, the samples of these studies were based on either certain one province [12] or bearing-age females of several provinces [13] among 31 provinces and autonomous regions of Chinese Mainland. Another study based on the 2002 China National Nutrition and Health Survey found that fetal, early-child and mid-child severely famine exposure were significant increased the risk of hypertension, but the age was not adjusted in statistical analyses [11]. All the exposed groups were older than non-exposed group, and thus the effect of age on hypertension cannot be excluded. Because of those limits in previous studies, further studies to explore the association between early life famine exposure and hypertension are in need.

In the present study, we used data from the 2011 China Health and Retirement Longitudinal Study (CHARLS) to examine the associations between famine exposure at fetal, infant and preschool stages and the risk of hypertension in later life, and we also explored whether a nutritionally rich conditions in later life modifies these associations.

## Methods

### The CHARLS national survey

The CHARLS is a large epidemiological survey of the elderly through randomly selecting the household which has at least one member who is 45 years old and above among 28 provinces, autonomous regions and municipalities of Chinese Mainland. The aims of the investigation are to analyze the dynamics of retirement and its interactions with health, health insurance and economic well-being, and to meet the needs of aging research. The CHARLS baseline survey was conducted from June 2011 to March 2012 through a face-to-face household interview and anthropometry by trained medical students in 28 provinces, autonomous regions and municipalities of Chinese Mainland. The baseline survey provides comprehensive and detailed information on demographics, socio-economic status, biomedical measurements and health status and functioning indicators, including blood pressure and hypertension, allowing us to estimate hypertension prevalence among four birth cohorts and different areas [14]. In order to ensure the representativeness of the sample, CHARLS baseline survey covered 450 villages nationwide, 150 counties and districts, eventually visiting 17,708 individuals in 10,257 households to represent the overall elderly population in China. The response rate was 80.51 % in all eligible households [14].

### Famine cohorts and areas

We categorized participants into four different exposure cohorts: non-exposed cohort, fetal-exposed cohort, infant-exposed cohort, and preschool-exposed cohort. All the cohorts were defined according to the participants’ birth dates. Participants who were born between October 1st, 1962, and September 30th, 1964, were classified as the non-exposed cohort. Participants who were born between October 1st, 1959, and September 30th, 1961, were classified as fetal-exposed cohort. Participants who were born between January 1st, 1958, and December 31st, 1958, were classified as infant-exposed cohort and participants born between January 1st, 1956, and December 31st, 1957, were classified as preschool-exposed cohort. Mean ages (standard deviation) of participants in non-exposed cohort, fetal-exposed cohort, infant-exposed cohort, and preschool-exposed cohort were 46.78(0.41), 50.41(0.62), 52.54(0.50), and 54.30(0.70) years, respectively.

The China Great Famine affected the whole mainland China, but the severity of famine sharply fluctuated across regions due to different climate conditions, population density, and local policies on food shortage [15]. As with previous studies, we also selected the excess death rate of every province as a indicator to determine the severity of the famine [15]. The excess death rate was calculated as the percentage change in mortality rate from the mean level in 1956–1958 years to the highest value during the period 1959–1961 years [15]. In the present study, an excess death rate of 50 % was used as the cutoff. Regions that had an equal or higher rate than the threshold were categorized as severely affected famine areas, and else categorized as the less severely affected famine areas. We split each of the four cohorts into severely affected famine areas and less severely affected famine areas. Therefore, with the four birth cohorts and two types of areas, it enabled us to examine the hypothesis that the health adverse effect is stronger in the severely affected famine areas than that in the less severely affected famine areas and to explore the effect on hypertension in both different birth cohorts and regional disparities.

### Blood pressure measures and variables

After at least a 10-min rest, blood pressure (BP) was measured on the participant’s left arm in seated position to the nearest mmHg according to standard procedures by trained medical students, requiring the arm is placed in the same height with heart, measuring three times at least 45 s apart during daytime (morning or afternoon) on the date of study interview; the average BP was regarded as a continuous outcome to measure. The diagnosed standard of hypertension was average diastolic blood pressure (DBP) ≥90 mmHg or systolic blood pressure (SBP) ≥140 mmHg [16] with three times measurements, or a previous diagnosis of hypertension by a registered clinician, or regular use of anti-hypertensive medication.

The bare height of participants was corrected to the centimeter and fasting body weight was corrected to 0.1 kg (kg). Both the height and weight were measured twice and the average values were used to calculate the body mass index (BMI) (BMI = weight/height^2^, kg/m^2^). We used the criteria recommended for Chinese adults and classified participants as overweight group if BMI ≥ 24 kg/m^2^, and else as normal group [17]. Overweight partly indicates to catch-up growth in a nutrient-rich environment in later life.

Current family economic status was assessed through the mean annual income in the year prior to the 2011 CHARLS baseline survey. The mean level of the 2,000 Chinese yuan per person per year was used as a cutoff point to classify all the subjects into high and low economic status groups.

Co-variables in current study included BMI, gender, smoking (yes/no) and drinking (at least once a month, yes/no). Ethnicity was not considered because almost all the subjects were Chinese Han population (the overwhelmingly dominant ethnicity group in China). Birth weight and gestational age at delivery were unavailable in the present study.

### Participants and samples

**Fig. 1:**
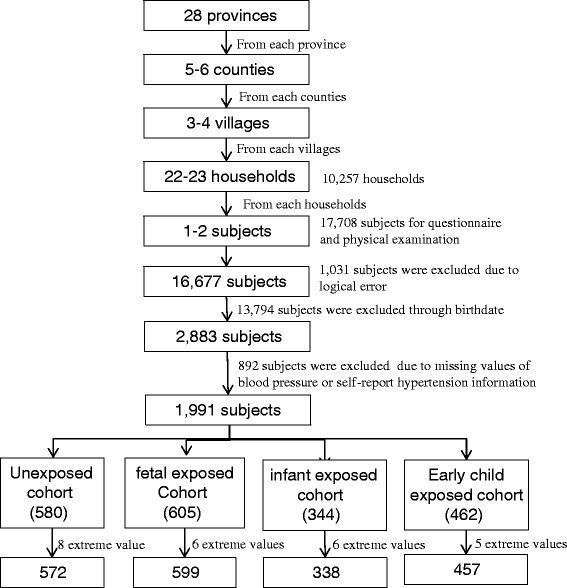
Flowchart on the sample of selecting methods in each step

Firstly, we selected 2,883 cases from public databases of the CHARLS 2011 baseline survey that collected 17,314 cases in 10,257 households through the birthdates of four cohorts defined above. Second, we excluded 892 cases due to missing values of blood pressure or self-report hypertension information. Further we excluded 25 cases with extreme blood pressure values (> five standard deviations of the SBP/DBP mean). Finally, 572 participants were enrolled in the non-exposed cohort, 599 participants in the fetal-exposed cohort, 338 participants in the infant-exposed cohort, and 457 participants in the preschool-exposed cohort, respectively. Therefore, a total of 1,966 participants were enrolled in the final analysis in present study (Fig. 1).

### Statistical analysis

We performed survey analysis with SPSS 20.0 for windows to estimate statistics data for this complex, multistage-designed survey sample. Data derived from the CHARLS 2011 baseline survey. A *P* < 0.05 was considered statistically significant.

AVONA and ANCOVA with BP as a dependent variable and age + BMI as covariates were conducted to test the difference of BP among four birth cohorts and multiple comparison between exposed cohorts and non-exposed, respectively.

The risk of hypertension among fetal-, infant- and preschool-exposed cohorts compared with non-exposed cohort were examined with the method of maximum likelihood by using binary logistic regression model in general areas adjusted for age, BMI, gender, smoking, and drinking.

Risks of hypertension among fetal-, infant- and preschool-exposed cohorts compared with non-exposed cohort were examined with the method of maximum likelihood by using binary logistic regression model. Interaction between famine exposure cohort (fetal, infant or preschool exposed vs. non-exposed) and area (less severely affected and severely affected) was tested by adding a multiplicative factor in the binary logistic regression model. Analyses were adjusted for gender, current smoking, and drinking. All the analysis was adjusted for age.

To examine whether the associations between early-life exposure to severe famine and hypertension was affected by a greatly improved nutritional environment in later life, we subsequently stratified the analyses by current family economic status and BMI in adulthood. Prevalence of hypertension was plotted according to cohort and classification of the stratification factors. The odds ratio of hypertension in the fetal-, infant- and preschool-exposed cohort compared with the non-exposed cohort was calculated within each category of the stratified factor.

## Results

Basic characteristics of the study subjects are shown in Table 1. In all the 1,966 subjects, 599 (30.5 %) participants had been exposed to the Chinese Great Famine in fetal period, 338 (17.2 %) participants had been exposed in infant period, and 457 (23.2 %) participants had been exposed in preschool period. BMI of infant-exposed and preschool-exposed cohorts was significantly higher than non-exposed cohort (*P* < 0.05). Compared with non-exposed cohort, SBP was significantly higher in infant-exposed and preschool-exposed cohorts (*P* < 0.05). No significant difference was observed for DBP (*P* > 0.05). Interesting, we observed an increased trend with age in smoking rate of participants (*P* < 0.05).

**Table 1:** Basic characteristics of study population according to Chinese famine exposure

Variables	Non-exposed cohort 10/1/1962-9/30/1964 (*n* = 572)	Fetal-exposed cohort 10/1/1959-9/30/1961 (*n* = 599)	Infant-exposed cohort 1/1/1958-12/31/1958 (*n* = 338)	Preschool-exposed cohort 1/1/1956-12/31/1957 (*n* = 457)
Gender n (%)				
Male	275(48.1)	282(47.2)	176(52.1)	236(51.6)
Female	297(51.9)	316(52.8)	162(47.9)	221(48.4)
Severity n (%)				
Severely	299(52.3)	317(52.9)	174(51.5)	250(54.7)
Less severely	273(47.7)	282(47.1)	164(48.5)	207(45.3)
Smoking n (%)^b^				
YES	214(37.4)	223(37.3)	143(42.4)	212(46.4)
NO	358(62.6)	375(62.7)	194(57.6)	245(53.6)
Drinking				
YES	86(15.0)	100(16.7)	57(16.9)	81(17.7)
NO	486(85.0)	499(83.3)	281(83.1)	376(82.3)
Age mean(SD) years^b^	46.78(0.41)	50.41(0.62)^d^	52.54(0.50)^d^	54.30(0.70)^d^
BMI mean(SD) kg/m^2a^	24.75(3.85)	24.61(4.14)	24.00(3.46)^c^	23.78(3.65)^d^
SBP mean(SD) mmHg^b,e^	125.19(17.36)	127.90(20.18)	131.45(19.41)^d, f^	129.01(20.00)^c, f^
DBP mean(SD) mmHg	77.86(12.81)	77.95(12.70)	79.06(12.89)	77.47(12.55)

^a^Mean values were significantly different among four birth cohorts (AVONA or *χ* ^2^ trend test; *P* < 0.01)

^b^Mean values were significantly different among four birth cohorts (AVONA or *χ* ^2^ trend test; *P* < 0.001)

^c^Mean values were significantly different between exposed cohorts and non-exposed cohort (Dunnet-*t* test, *P* < 0.05)

^d^Mean values were significantly different between exposed cohorts and non-exposed cohort (Dunnet-*t* test, *P* < 0.01)

^e^Mean values were significantly different by ANCOVA with BP as a dependent variable and age + BMI as covariates among four birth cohorts (*P* < 0.05)

^f^Mean values were significantly different by ANCOVA with BP as a dependent variable and age + BMI as covariates between exposed cohorts and non-exposed cohort (*P* < 0.05)

Table 2 presented the prevalence rates of hypertension among three exposed cohorts and non-exposed cohort. Compared with non-exposed cohort, the prevalence of hypertension in the non-exposed, fetal-exposed, infant-exposed and preschool exposed cohorts was 18.9, 20.7, 28.7, and 23.4 %, respectively. Only in infant-exposed cohort, the risk of hypertension was significantly higher (OR 1.71; 95 % CI 1.14, 2.56; *P* = 0.009) than non-exposed cohort, even after adjusted BMI, gender, smoking and drinking (OR 1.66; 95 % CI 1.04, 2.66; *P* = 0.036).

**Table 2:** Hypertension prevalence and risks of three exposed cohorts compared with non-exposed cohort

Variables	Non-exposed cohort	Fetal-exposed cohort	Infant-exposed cohort	Preschool-exposed cohort
Hypertension				
Prevalence (%)	18.9	20.7	28.7	23.4
*P* ^a^		0.822	0.009	0.214
Odds ratio (95 % CI)^a^	Ref.	0.93(0.50–1.75)	1.71(1.14–2.56)	1.29(0.86–1.92)
*P* ^b^		0.811	0.036	0.235
Odds ratio (95 % CI)^b^	Ref.	0.92(0.45–1.88)	1.66(1.04–2.66)	1.33(0.83–2.14)

All the analysis was adjusted for age

^a^Evaluating the overall risk of three exposed cohort with non-exposed as reference by single variance binary logistics regression model

^b^Evaluating the risk of three exposed cohorts with non-exposed as reference by multi-variance binary logistics regression model after adjusted for BMI, gender, smoking and drinking

The results of multivariate logistics regressions to evaluate the risk of famine exposed cohorts stratified by areas and cohorts are shown in Table 3. In less severely affected famine areas, compared with non-exposed cohort, the crude and adjusted risk of hypertension for three exposed cohorts had no statistically significant difference (*P* > 0.05). However, in severely affected famine area, compared with non-exposed cohort, only the odds ratio of hypertension for infant-exposed cohort was statistically significant (OR 2.12; 95 % CI 1.19, 3.79; *P* = 0.011), even after adjusting gender, smoking, and drinking (OR 2.11; 95%CI 1.18, 3.77; *P* = 0.012). But in fetal-exposed and preschool exposed cohorts, both the crude and adjusted risks of hypertension had no statistically significant difference compared with non-exposed cohort (*P* > 0.05). A significant interaction between the exposed cohorts and areas were found only in the infant exposed cohort both crude prevalence of hypertension (*P* = 0.001) and after adjusted covariance (*P* = 0.009).

**Table 3:** Hypertension prevalence and risks of three exposed cohorts in severely and less severely affected areas compared with non-exposed cohort

	Non-exposed cohort	Fetal-exposed cohort	Infant-exposed cohort	Preschool-exposed cohort
Severely affected famine area				
Prevalence (%)	19.4	20.8	30.5	25.6
*P* ^a^		0.998	0.011	0.059
Odds ratio (95 % CI)^a^	Ref.	1.00(0.39–2.57)	2.12(1.19–3.79)	1.73(0.98–4.06)
*P* ^b^		0.989	0.012	0.069
Odds ratio (95 % CI)^b^	Ref.	0.99(0.39–2.56)	2.11(1.18–3.77)	1.70(0.96–3.01)
Less severely affected famine area				
Prevalence (%)	18.3	20.6	26.8	20.8
*P* ^a^		0.743	0.261	0.796
Odds ratio (95 % CI)^a^	Ref.	0.88(0.37–2.03)	1.38(0.79–2.41)	1.17(0.74–1.84)
*P* ^b^		0.763	0.248	0.906
Odds ratio (95 % CI)^b^	Ref.	0.88(0.37–2.06)	1.40(0.79–2.46)	0.97(0.54–1.72)
*P* for interaction between area and cohort^a^	Ref.	0.459	0.001	0.144
*P* for interaction between area and cohort^b^	Ref.	0.901	0.009	0.062

All the analysis was adjusted for age

^a^Evaluating the overall risk of three exposed cohort with non-exposed as reference by single variance binary logistics regression model

^b^Evaluating the risk of three exposed cohorts with non-exposed as reference by multi-variance binary logistics regression model after adjusted for gender, smoking and drinking

**Fig. 2:**
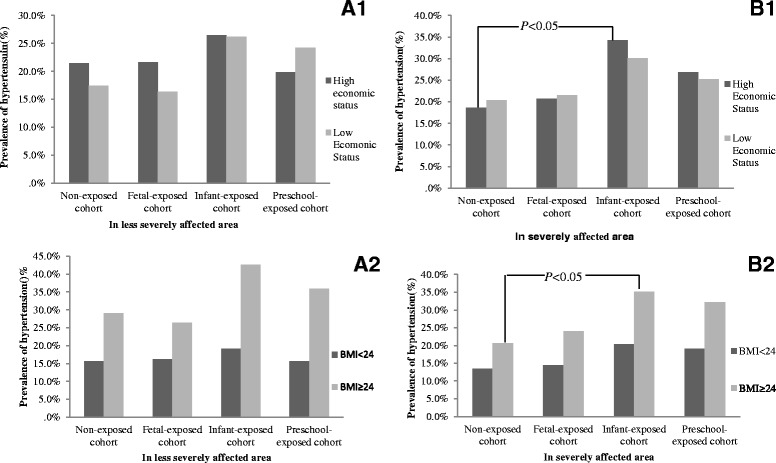
Stratified analyses by economic status, and BMI for less severely and severely famine affected areas. **A1** and **B1** present the difference of hypertension prevalence between high economic and low economic status groups in less severely and severely affected areas, respectively. **A2** and **B2** present the difference of hypertension prevalence between BMI ≥ 24.0 kg/m^2^ and BMI < 24.0 kg/m^2^ in less severely and severely affected areas, respectively

Stratified analyses by economic status, and BMI for less severely and severely famine affected areas are shown in Fig. 2 and Table 4. Figure 2 A1 and B1 showed only in severely affected areas, the hypertension risk of infant-exposed cohort was significant higher (OR 2.26; 95 % CI 1.19, 4.31; *P* = 0.013) than non-exposed cohort in high economic status group, even after adjusted gender, smoking, and drinking (OR 2.18; 95 % CI 1.14, 4.18; *P* = 0.019). We have not observed consistent associations in less severely affected area, low economic status group and other cohorts in severely affected area (*P* > 0.05).

**Table 4:** Prevalence rate of hypertension by economic status, birth cohort and severity of the Chinese famine area

Variables	Non-exposed cohorts	Fetal-exposed cohorts	Infant-exposed cohort	Preschool-exposed cohort
Low economic status				
Severely affected famine area				
Prevalence (%)	20.3	21.6	30.1	25.2
*P* ^a^		0.805	0.102	0.368
Odds ratio (95 % CI)^a^	Ref.	1.08(0.60–1.95)	1.69(0.90–3.18)	1.52(0.72–2.45)
*P* ^b^		0.783	0.104	0.346
Odds ratio (95 % CI)^b^	Ref.	1.09(0.60–1.98)	1.69(0.90–3.19)	1.35(0.73–2.49)
Less severely affected famine area				
Prevalence (%)	17.4	16.3	26.2	24.2
*P* ^a^		0.832	0.176	0.277
Odds ratio (95 % CI)^a^	Ref.	0.92(0.45–1.92)	1.68(0.79–3.58)	1.52(0.72–3.21)
*P* ^b^		0.678	0.19	0.300
Odds ratio (95 % CI)^b^	Ref.	0.86(0.41–1.79)	1.67(0.78–3.58)	1.49(0.70–3.19)
*P* for interaction between area and cohort^a^	Ref.	0.325	0.609	0.883
*P* for interaction between area and cohort^b^	Ref.	0.341	0.635	0.956
High economic status				
Severely affected famine area				
Prevalence (%)	18.7	20.8	34.2	26.8
*P* ^a^		0.654	0.013	0.119
Odds ratio (95 % CI)^a^	Ref.	1.14(0.64–2.04)	2.26(1.19–4.31)	1.60(0.89–2.88)
*P* ^b^		0.756	0.019	0.187
Odds ratio (95 % CI)^b^	Ref.	1.10(0.61–1.97)	2.18(1.14–4.18)	1.50(0.82–2.71)
Less severely affected famine area				
Prevalence (%)	21.4	21.7	26.5	19.8
*P* ^a^		0.962	0.387	0.751
Odds ratio (95 % CI)^a^	Ref.	1.01(0.58–1.76)	1.32(0.70–2.49)	0.91(0.50–1.66)
*P* ^b^		0.866	0.403	0.788
Odds ratio (95 % CI)^b^	Ref.	1.05(0.60–1.83)	1.31(0.69–2.48)	0.92(0.50–1.69)
*P* for interaction between area and cohort^a^	Ref.	0.845	0.291	0.198
*P* for interaction between area and cohort^b^	Ref.	0.69	0.304	0.179

^a^Evaluating the overall risk of three exposed cohort with non-exposed as reference by single variance binary logistics regression model

^b^Evaluating the risk of three exposed cohorts with non-exposed as reference by multi-variance binary logistics regression model after adjusted for gender, smoking and drinking

Figure 2 B2 and Table 5 showed in severely famine affected area, the prevalence of hypertension was significantly higher for subjects who had BMI ≥ 24 kg/m^2^ than BMI < 24 kg/m^2^ in all birth cohorts. Compared with non-exposed cohort, the risk ratio of hypertension in infant-exposed cohort was 1.65 (95 % CI 0.71, 3.83; *P* = 0.246) for those who BMI <24 kg/m^2^, and 2.09 (95 % CI 1.09, 4.01; *P* = 0.026) in subjects with BMI ≥ 24 kg/m^2^. Similar analyses were performed in subjects who exposed in less severely affected famine areas (A2), but no statistically significant difference was observed (*P* > 0.05).

**Table 5:** Prevalence rate of hypertension by BMI, birth cohort and severity of the Chinese famine area

Variables	Non-exposed cohorts	Fetal-exposed cohorts	Infant-exposed cohort	Preschool-exposed cohort
BMI < 24.0 kg/m^2^				
Severely affected famine area				
Prevalence (%)	13.4	14.4	20.3	19.0
*P* ^a^		0.833	0.246	0.280
Odds ratio (95 % CI)^a^	Ref.	1.09(0.50–2.39)	1.65(0.71–3.83)	1.52(0.71–3.25)
*P* ^b^		0.910	0.284	0.351
Odds ratio (95 % CI)^b^	Ref.	0.95(0.43–2.14)	1.59(0.68–3.74)	0.44(0.67–3.10)
Less severely affected famine area				
Prevalence (%)	15.6	16.2	19.2	15.6
*P* ^a^		0.913	0.545	1.000
Odds ratio (95 % CI)^a^	Ref.	1.04(0.49–2.22)	1.28(0.58–2.86)	1.00(0.46–2.18)
*P* ^b^		0.923	0.552	0.987
Odds ratio (95 % CI)^b^	Ref.	1.04(0.48–2.24)	1.28(0.57–2.89)	0.99(0.45–2.20)
*P* for interaction between area and cohort^a^	Ref.	0.717	0.868	0.523
*P* for interaction between area and cohort^b^	Ref.	0.93	0.733	0.46
BMI ≥ 24.0 kg/m^2^				
Severely affected famine area				
Prevalence (%)	20.6	24.0	35.2	32.1
*P* ^a^		0.53	0.026	0.062
Odds ratio (95 % CI)^a^	Ref.	1.21(0.66–2.21)	2.09(1.09–4.01)	1.82(0.97–3.42)
*P* ^b^		0.674	0.029	0.090
Odds ratio (95 % CI)^b^	Ref.	1.14(0.62–2.09)	2.07(1.08–3.98)	1.73(0.92–3.28)
Less severely affected famine area				
Prevalence (%)	29.0	26.4	42.6	35.8
*P* ^a^		0.681	0.112	0.395
Odds ratio (95 % CI)^a^	Ref.	0.88(0.47–1.64)	1.81(0.87–3.76)	1.37(0.67–2.80)
*P* ^b^		0.833	0.097	0.306
Odds ratio (95 % CI)^b^	Ref.	0.93(0.49–1.77)	1.88(0.89–3.97)	1.48(0.70–3.10)
*P* for interaction between area and cohort^a^	Ref.	0.671	0.422	0.655
*P* for interaction between area and cohort^b^	Ref.	0.559	0.823	0.539

^a^Evaluating the overall risk of three exposed cohort with non-exposed as reference by single variance binary logistics regression model

^b^Evaluating the risk of three exposed cohorts with non-exposed as reference by multi-variance binary logistics regression model after adjusted for gender, smoking and drinking

## Discussion

In this study, we observed that compared with non-exposed cohort, the risk of hypertension was significantly higher for infant -exposed cohorts in severely affected areas, compared with non-exposed cohort, and the risk was higher in participants with BMI ≥ 24 kg/m^2^ in adulthood than participants with BMI < 24 kg/m^2^. For other famine exposed cohorts, consistent association was not observed in the current study.

Several mechanisms might explain the associations between exposure to famine in infanthood and the risk of hypertension in adulthood. 1) Exposure to extremely malnutrition might lead to low birth weight which has been shown to be associated with a reduction of nephrons in numbers which limits kidney’s ability to excrete sodium and raises blood pressure in adulthood [18, 19]. Furthermore, reduced nephron endowment exacerbates the hypertensive effects of obesity [20]. The postnatal environment with rich nutrient that mismatches with infant-period environment under extreme malnutrition might elevate the risk of hypertension in adulthood. The associations between early-life exposed famine and risk of hypertension were stronger among subjects who became overweight in later life in the current study. 2) Experience of famine during infanthood may alter the expression of the renin-angiotensin system (RAS), and subsequently alter the renal vascular and tubular structures, and increase the risk of hypertension in adulthood. Animal studies have demonstrated that, in animals with intrauterine growth restriction due to nutrition deficiency, angiotensin II receptors were highly expressed in the kidneys of newborn piglets and rat [21–24]. As a major regulator of blood pressure control and volume homeostasis, the RAS plays a very important role in the pathogeny of hypertension [21–24]. Furthermore, incommensurate activation of the peripheral RAS, had been validated by a significant increase in plasma renin activity, which exists after development of hypertension [24, 25]. More importantly, hypertension is abolished by systemic blockade of the RAS [24, 25] indicating that the RAS contributes to hypertension programmed in response to maternal nutrient restriction [26]. Several researches have also showed that stress induced by famine in fetal period could activate a complex range of responses which involve endocrine, nervous, and immune systems [27]. Appropriate regulation of stress responses has been linked to hypertension [19]. 3) Our finding is further supported by genetic research. Pham and his colleagues showed that exposure to extreme undernutrition might alter the methylation level of p53 gene, affect mRNA levels of critical apoptosis-related proteins, increase renal apoptosis, and reduce nephron number in the intrauterine growth retardation kidney [28].

To our knowledge, three great famines in the nineteenth century have explored the relationship between early life exposed to famine and hypertension risk in later life. Several Dutch and Leningrad Siege famine studies found inconsistent associations concerning the effect of fetal stage exposed to famine on BP or hypertension in later life. Stein AD et al. [7] and Roseboom T et al. [6] observed a significant association, but Stanner SA [9], de Rooij S R and colleagues [8] and Roseboom TJ [29] did not observed the consistent association. We speculate that these conflicting results between Dutch and Leningrad Siege famine studies could be caused by variable accuracy to define the exposure and different nutrient condition exposures in extrauterine environment. The Dutch population who were exposed to famine could restore from the adverse effect when rapidly turning into a nutritionally rich environment after the end of famine, while the population that exposed to Leningrad famine remained a relatively poor nutritional conditions and lasted for a longer time. It was proved by an animal model [19], showing that fewer nephron numbers might be a link between low birth weight and hypertension in adulthood, but a rich postnatal feeding conditions might restore the nephron capacity and prevent the later-life development of hypertension [30].

Three studies have reported the association between early-life China famine exposure and risk of hypertension. Analyzing data of 7,874 participants from the 2002 China National Nutrition and Health Survey, Li and her colleagues [11] observed a marginally increased risk of hypertension (OR 1.88, 95 % CI: 1.00 3.53, *P* = 0.05) in fetal-period exposed cohort in severely affected areas compared with the control cohort of subjects born from 1st October 1962 through 30th September 1964. Although the ranges of birthdates in Li’s study were the same both in fetal exposed group and control group as the current study, the association was inconsistent with the current study (OR 1.10; 95 % CI 0.67, 1.78; *P* = 0.712). Interestingly, our study observed a significant moderate association (OR 2.11; 95 % CI 1.18, 3.77; *P* = 0.012) between hypertension and infant famine exposed cohort in severely affected areas, but we could not compare the results with Li’s study since two studies had different birthdate ranges in infant-exposed group. In a study of 12,065 adults born 1957–1964 in the Zhongshan and Nanhai cities of Guangdong province, China, belonging to the less severely affected areas based on the classification in this study, Wang observed 1.83 times higher in those exposed only during infancy (adjusted OR 1.83; 95 % CI 1.61, 2.08; *P* < 0.05) as compared with non-exposed subjects, which was inconsistent with the current study (adjusted OR 1.40; 95 % CI 0.79, 2.46; *P* = 0.246). Furthermore, participants in Wang’s study were selected only from one province of China. Another study found a 3-fold increase the risk of hypertension for bearing-age female who infant exposure famine in three provinces of China mainland [13], which was consistent with the current study. However, compared with the current study, the latest study mentioned before might lack the broad representativeness of the whole mainland of China.

Our study used threshold, BMI ≥ 24.0 kg/m^2^, and 2,000 Chinese yuan per person per year as a cutoff point to categorize overweight and family economic status, respectively. Overweight and high economic status may partly stand for a “rich” nutrient environment in China. The current study observed that compared with subjects with BMI < 24.0 kg/m^2^ or low economic status, overweight (BMI ≥ 24.0 kg/m^2^) or high economic status group in infant-exposed cohort had a higher hypertension prevalence rate and risk in later life. The similar results were reported in several studies [11, 31–33]. The study of Li also found the associations between early-life exposure to famine and hypertension risk were stronger among subjects who became overweight in later life. Results of these studies indicated that the postnatal environment with rich nutrient that mismatches with infant-period environment of extreme malnutrition might elevate the risk of hypertension in adulthood.

We noted that hypertension was more prevalent in individuals with increased BMI. It is likely that metabolic syndrome may be prevalent in famine-exposed cohorts. Therefore, insulin resistance may be a mediator of the association between famine and hypertension. However, the CHARLS study did not collect information on insulin resistance, and the impact of insulin resistance cannot be evaluated in the current study. Future studies on this topic are warranted.

The primary limitation of the current study is selection bias caused by excess mortality in early life. The Great Famine might leave the stronger and healthier subjects and get rid of participants that held abnormal metabolism and structure. This selection bias may decrease the risk of hypertension for exposed cohorts, indicating the selection bias could not enhance the associations in the current study. Second, participants who were exposed to famine in fetal period may also experience actual exposure in infant period partly due to China’s famine lasting three years (1959–1961). There was not a good method to distinguish accurately whether they were fetal-exposed or infant-exposed to famine. But in the current study, we defined infant-exposed cohort born from 1st January 1958 to 31st December 1958, which insured almost all the participants in this group were exposed to famine in infanthood. Third, we used the excess mortality as indirect indicator to measure the severity of famine exposure. With the method, we cannot only attribute the death to famine exposure, because bad weather conditions or infection could also cause the excess mortality. We also had no data of personal food supplement and total caloric change. Therefore, we cannot conclude that the higher risk of hypertension among cohorts exposed to famine is totally attributed to malnutrition in early life. In addition, as is similar to other China famine studies, our study lacks the objective indicators that evaluate the effect of famine exposure on health outcome, such as birth weight, body length, etc. Besides, hypertension diagnosed by a single office visit might be also a limitation in the current study. Although there were several limitations, our study used a national broadly representative data, observing infant exposed to famine that lasted much longer and affected more people in China elevated the risk of hypertension in later life. And a rich nutrient environment in postnatal increased the risk of hypertension.

## Conclusions

We found that exposure to severely China’s famine in infancy increased the risk of hypertension in later life. And a nutritional rich postnatal environment further increased the risk. Our study showed that early life conditions were crucial for the risk of hypertension.
